# *Physcomitrium patens* (Hedw.) Mitt. growing in the dark defaults to an auxin- and cytokinin-independent developmental programme

**DOI:** 10.17912/micropub.biology.000915

**Published:** 2023-08-19

**Authors:** Shawn R Robinson, Ryan R McDonald, Neil W Ashton

**Affiliations:** 1 Biology, University of Regina, Regina, Saskatchewan, Canada

## Abstract

Auxin and cytokinin partially restore *Physcomitrium* (formerly *Physcomitrella* ) *patens* gametophores that have developed in the dark to a form more typical of those grown in light. Auxin synthesis and/or transport in gametophores decrease with time spent in the dark. Auxin synthesis resumes in the apices of dark-grown gametophores upon their return to the light. Red light and to a lesser extent blue light are sufficient for this. The *mas* and *GH3* promoters are both auxin-inducible but respond differentially to spatial cues.

**Figure 1. Physcomitrium skotomorphogenesis and photomorphogenesis:**
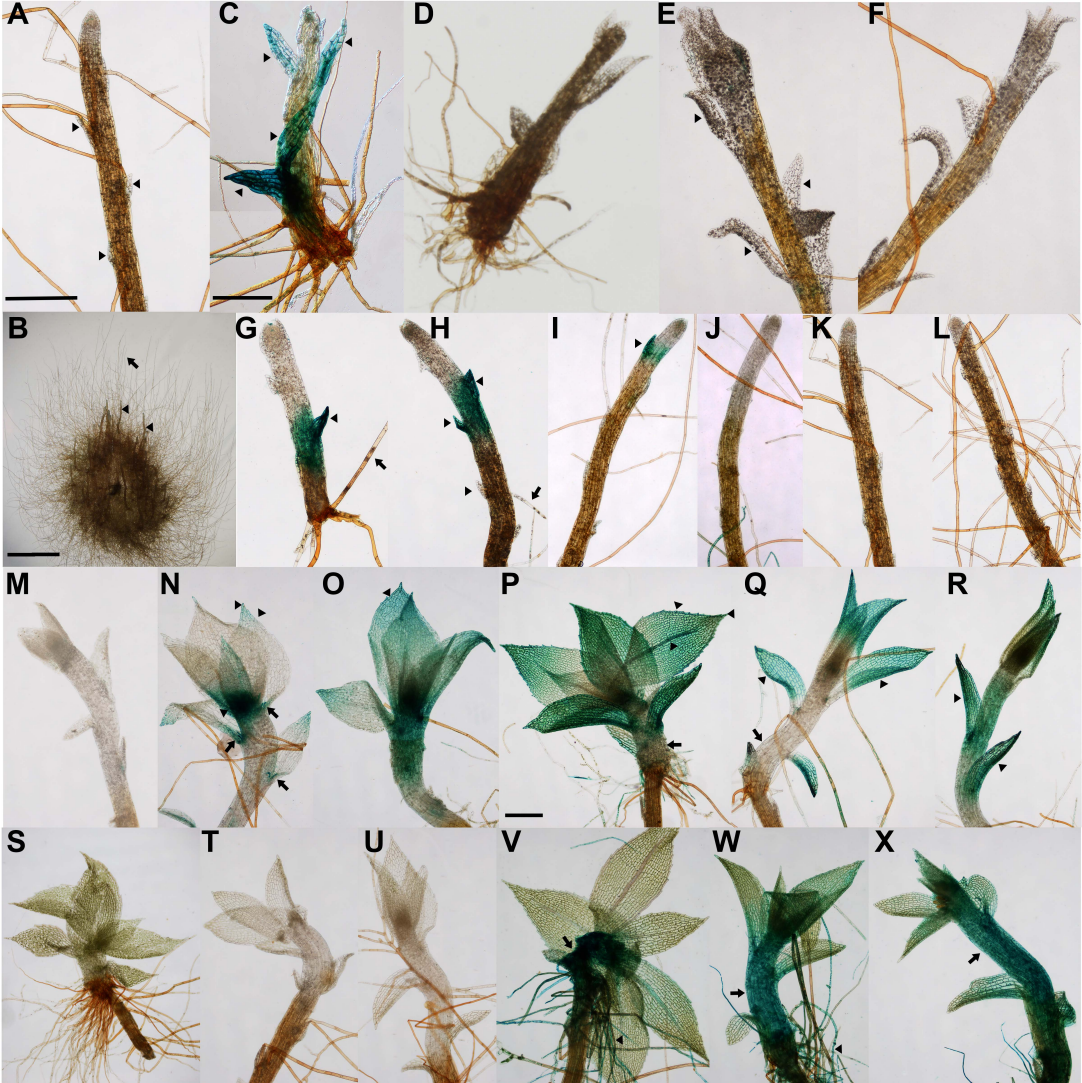
**(A)** Gametophore of LTII-2 cultured for 21 d in WL, followed by 21 d in the dark on fresh medium containing 0.5% sucrose. Arrowheads indicate scale-like leaves. **(B)** Whole gametophytic colony of *pabB4* cultured for 21 d in WL, followed by 21 d in the dark. Arrowheads indicate etiolated, negatively gravitropic gametophores that have developed in the dark. Arrow indicates negatively gravitropic, dark-grown peripheral caulonemata lacking gametophores. **(C)** Gametophore of LTII-2 cultured for 21 d in WL, followed by 21 d in the dark on fresh medium containing 50 nM NAA. Arrowheads indicate partially expanded leaves expressing GUS (blue staining). **(D)** Gametophore of *pabB4* cultured for 21 d in WL, followed by 21 d in the dark on medium containing 50 nM NAA. **(E)** Gametophore of LTII-2 cultured for 21 d in WL, followed by 21 d in the dark on medium containing 100 nM BAP. Arrowheads indicate unstained, partially expanded leaves. **(F)** Gametophore of *pabB4* cultured for 21 d in WL, followed by 21 d in the dark on medium containing 100 nM BAP. **(G, H)** Gametophores of LTII-2 cultured for 21 d in WL, followed by 6 d or 9 d respectively in the dark. Arrowheads indicate GUS-expressing (blue) non-apical scale-like leaves. Arrows point to rhizoids with punctuated blue staining. There also appears to be GUS expression in the non-apical region of the gametophore stems. **(I, J)** Gametophores of LTII-2 cultured for 21 d in WL, followed by 14 d in the dark. Arrowhead indicates a single GUS-expressing (blue) scale-like leaf. There is no blue staining in the gametophore stems. **(K)** Gametophore of LTII-2 cultured for 21 d in WL, followed by 21 d in the dark. There is no blue staining in gametophore stem or scale-like leaves. **(L)** Gametophore of *pabB4* cultured for 21 d in WL, followed by 21 d in the dark. **(M)** Gametophore of LTII-2 cultured for 21 d in WL, followed by 21 d in the dark after which it was returned to WL for 48 h. There is no blue staining in the gametophore stem or in the partially expanded leaves. **(N, O)** Gametophores of LTII-2 cultured for 21 d in WL, followed by 21 d in the dark after which they were returned to WL for 72 h. Arrowheads indicate blue staining in apical and marginal cells and distal laminal regions of expanded leaves, and in young leaf primordia in the gametophore apex. Arrows point to stained mucilaginous hairs in the axils of lower leaves. **(P, Q, R)** Gametophores of LTII-2 cultured for 21 d in WL, followed by 21 d in the dark after which they were returned to WL, RL or BL respectively for 7 d. Arrowheads indicate blue staining in the apical and marginal cells and midrib of fully expanded leaves exposed to WL and blue-stained leaves exposed to RL or BL. The laminae of leaves exposed to WL are turquoise resulting from the combination of blue staining (GUS expression) and light-driven synthesis of photosynthetic pigments. Arrows point to thickened, unstained, light-grown gametophore stems. **(S, T, U)** Gametophores of *pabB4* cultured for 21 d in WL, followed by 21 d in the dark after which they were returned to WL, RL or BL respectively for 7 d. **(V, W, X)** Gametophores of GH3::GUS cultured for 21 d in WL, followed by 21 d in the dark after which they were returned to WL, RL or BL respectively for 7 d. Arrows indicate darkly stained and thickened region of the gametophore stems developed in the light. Arrowheads indicate darkly blue-stained rhizoids. Scale bars represent 250 µm **(A, C, P)** and 5mm **(B)** .

## Description

In 1979, Ashton et al. constructed a model describing the roles and interactions of auxin and cytokinin in *Physcomitrium* photomorphogenesis from spore germination to leafy gametophore formation. Since then, this model has been elaborated incorporating interactions of these phytohormones with light (Ashton et al. 1990a and b). In 1988 at the Plant Growth Substances conference held in Calgary, Ashton et al. first proposed that skotomorphogenesis in *Physcomitrium* follows a default developmental programme that is independent of auxin and cytokinin. This contention was based primarily on the observation that various categories of auxin- and/or cytokinin-insensitive mutants, which are also characterised by the overproduction of chloronemata and the absence or underproduction of caulonemata and gametophores when grown in the light, resembled the wild-type line when grown in the dark, whereupon the proliferation of chloronemata ceases and the chloronemal apical cells differentiate into caulonemal apical cells that divide and produce negatively gravitropic caulonemata. They concluded that the differentiation of primary chloronemal cells into caulonemal apical cells may be light-independent, at least under some circumstances, and in darkness this transition may require no, or only very low levels, of auxin and cytokinin. They also suggested that a significant role of these two hormone types is to antagonise a light-promoted maintenance and proliferation of chloronemal apical cells. Here we provide further evidence for our original hypothesis using a stable transgenic line, LTII-2, possessing genomically integrated pBDH001 cII. This plasmid contains the mannopine synthase ( *mas* ) 1’, 2’ dual promoter with the 2’ end fused to the *E. coli* β-glucuronidase reporter gene ( *uidA* ). The 2’ end of this auxin-inducible promoter supports approximately seven- to eight-fold greater activity than the 1’ end (Leung et al. 1991) .

In light-grown *Physcomitrium* LTII-2 stained for β-glucuronidase, the strongest GUS expression was present in the leaf apical and marginal cells as well as in the leaf lamina, especially at the apical ends of young leaves. Little or no activity was detected in gametophore stems and rhizoids or in leaves that had attained their final size. A few caulonemal cells and SBIs were stained but the majority were not (McDonald 1999) . GUS activity in young apical leaves began at the apex and progressed basipetally to the leaf base (Robinson 2015) in sync with a basipetal wave of PIN expression (Viaene et al. 2014) and cell expansion (Barker and Ashton 2013) . We conclude that auxin is transported basipetally in developing leaves and is responsible for promoting cell expansion. This pattern of *GUS* expression differs from that reported in *Physcomitrium* using an auxin-inducible soybean *GH3* promoter fused to the *uidA* gene. In this case, the whole gametophore stem was stained with maxima at the apex and base. However, in the absence of induction with exogenous auxin, leaves were never stained. Protonemal staining was weak but evenly distributed (Bierfreund et al. 2003). We suggest that these auxin-inducible promoters are differentially responsive to spatial cues within developing plants.

When light-grown colonies were transferred to the dark, chloronemal apical cells differentiated into caulonemal apical cells and together with existing caulonemal apical cells divided to produce negatively gravitropic caulonemal filaments. Existing buds differentiated into negatively gravitropic, etiolated gametophores with regularly spaced, small scale-like leaves appressed to the gametophore stems ( Fig. 1A and B ). LTII-2 resembled *pabB4* , the non-transgenic strain we used as a control line for comparison. To confirm LTII-2 is auxin-inducible in the dark, we cultured it in the presence of the synthetic auxin, 1-naphthalene acetic acid (NAA) ( Fig. 1C ). LTII-2 and *pabB4* ( Fig. 1D ) gametophores were morphologically identical with larger leaves than gametophores grown in the absence of NAA ( Fig. 1A ). The gametophores were also shorter. This partial restoration to a phenotype more typical of light-grown gametophores was accompanied by blue staining (indicative of GUS expression) of the leaves of LTII-2. LTII-2 and *pabB4* gametophores cultured in the presence of the synthetic cytokinin, 6-benzylaminopurine (BAP) ( Fig. 1E and 1F) were also characterised by the development of larger leaves but were not noticeably shorter than those grown in the absence of BAP. BAP did not induce GUS expression in LTII-2.We conclude that the *mas* promoter is specifically induced by auxin and not by cytokinin and that dark-grown gametophores either lack or possess lower levels of auxin and cytokinin.

Light-grown colonies transferred to the dark for different lengths of time revealed the progressive loss of *GUS* expression, indicative of decreasing endogenous auxin levels. Thus, blue staining was apparent after six and nine days in the dark, notably in the scale-like leaves and stem of non-apical regions of gametophores ( Fig. 1G and 1H). By fourteen days in the dark, only the occasional scale-like leaf was stained in some gametophores ( Fig. 1I ) but not all gametophores ( Fig. 1J ) and after 21 days, no staining was apparent ( Fig. 1K ) and gametophores of LTII-2 resembled those of *pabB4* ( Fig. 1L ).

Further evidence that auxin levels are absent or reduced in the dark was obtained by returning dark-grown LTII-2 and GH3::GUS cultures to white light (WL), red light (RL) or blue light (BL). Gametophores of LTII-2 colonies grown for 21 days in the dark, showed no blue staining after two subsequent days in WL ( Fig. 1M ). After three days in WL, GUS expression appeared in mucilaginous hairs, the gametophore apex and the apical and marginal cells and apical lamina of expanded leaves ( Fig. 1N and 1O). By day seven in WL, apical leaves were fully expanded and the whole leaf lamina was turquoise as a result of the combination of chlorophyll induction and blue staining from *GUS* expression. Leaf marginal cells and midribs were stained blue. Gametophore stems became thicker but remained unstained ( Fig. 1P ). Etiolation of the stem was abolished. These observations accord with those described earlier for light-grown LTII-2. After seven days in RL, mucilaginous hairs and partially expanded leaves were stained blue but there was no GUS expression in the thickened stem apex ( Fig. 1Q ). After seven days in BL, leaves were stained blue and pale blue staining was present in the upper part of the stem. Thickening of the gametophore stem did not occur in BL ( Fig. 1R ). No blue staining occurred in the *pabB4* control line ( Fig. 1S -U). Dark-grown GH3::GUS colonies returned to light behaved differently to LTII-2. After seven days in WL, the thickened gametophore stem and rhizoids were stained dark blue, but the lamina of expanded leaves was green and GUS expression was absent ( Fig. 1V ). Similarly, the region of the stem that had developed in RL or BL and rhizoids were stained blue, but the lamina of the partially expanded leaves remained green ( Fig. 1W and X ) with blue stain restricted to the leaf apical and marginal cells in RL ( Fig. 1 W ).

We interpret our observations as follows.

· Full leaf expansion, lateral expansion of the gametophore stem, abolition of etiolation and synthesis of photosynthetic pigments are light-driven processes.

· RL and BL are sufficient for partial restoration of leaf expansion and RL is sufficient for lateral expansion of the gametophore stem.

· Auxin and cytokinin are required for leaf expansion. Auxin and possibly cytokinin are needed for lateral expansion of the gametophore stem.

· Auxin and cytokinin synthesis are light-inducible.

· The auxin-inducible *mas* and *GH3* promoters are differentially responsive to spatial cues in developing *Physcomitrium* gametophytes.

· Skotomorphogenesis, including negative gravitropism of caulonemata and gametophores, requires no or lower levels of auxin and cytokinin than photomorphogenesis. Thus, in the dark *Physcomitrium* defaults to an auxin- and cytokinin-independent developmental programme. Bennett et al. (2014) have provided further support for a lack of involvement of auxin in the negative gravitropism of caulonemata by demonstrating that gravitropic behaviour in the dark is unaffected in double PIN ( *pinApinB* ) genetic disruptants. Contrastingly, gametophores of the *pinApinB* mutant line are agravitropic. Therefore, we conclude that the gravitropic response of gametophores requires a low level of auxin synthesis in the gametophore stem or of acropetal transport along the stem of auxin synthesised by light-grown tissues prior to their being transferred into the dark.

· A significant role of auxin and cytokinin is to counteract the light-driven promotion of chloronemata. This has received strong support from the finding that blue light, acting via cryptochromes, inhibits the transition of chloronemal apical cells into caulonemal apical cells, the differentiation of caulonemal side branch initials (SBIs) into secondary caulonemata and buds and the growth of gametophore stems, while promoting the formation of SBIs and their growth and division generating secondary chloronemata. Furthermore, cryptochrome signals inhibit auxin responses including the expression of auxin-inducible genes (Imaizumi et al. 2002) .

## Methods

Standard medium and culture conditions for growing *Physcomitrium* in continuous white light (WL) were as described previously (Rabbi et al. 2020) . When grown on medium containing adequate *p* -aminobenzoic acid (PABA: 1.8 µM and 18 µM for gametophytes and sporophytes respectively), the *pabB4* line used as a control for comparison with the transgenic lines, LTII-2 and GH3::GUS, is phenotypically indistinguishable from the original Gransden wild-type (obtained from a single spore isolated from nature by HLK Whitehouse in 1962) from which it was derived. In most experiments, gametophytes were cultured in Petri dishes for three weeks in WL prior to being transferred on to fresh medium containing 0.5% sucrose and placed in the dark. Transfer on to fresh medium was facilitated by using sterile, porous cellophane discs according to Grimsley et al. (1977). By this point, gametophytic colonies had produced caulonemata and gametophore buds, which had not yet developed into leafy shoots. Plates wrapped in aluminium foil were stacked vertically with the lids and bases parallel to the gravity vector. Cultures were incubated in the dark for up to three weeks after which some of them were returned to continuous WL (approx. 30 µmol m ^-2^ s ^-1^ at the surface of the medium), RL (0.95 µmol m ^-2^ s ^-1^ ) or BL (0.78 µmol m ^-2^ s ^-1^ ). Petri plates in WL were covered with one layer of clear resin filter (Roscolux No. 114, Hamburg Frost). RL and BL were achieved respectively by substituting the clear resin filter with a red filter (Roscolux No. 27, Medium Red) or a blue filter (Lee Filters No. 713J, Winter Blue).

Plasmid pBDH001 cII was introduced into Gransden 1962 wild-type *Physcomitrium* by particle bombardment-mediated transformation according to Sawahel et al. (1992). The transgenic line LTII-2 was selected by its resistance to hygromycin B (30 µg.ml ^-1^ ) and was classified as a stable transformant after retaining its resistance to hygromycin after five rounds of growth on non-selective medium. The presence of pBDH001 cII in LTII-2 was verified by PCR with the primer pair, HPT Upper plus HPT Lower, designed to detect the plant-active *nos* promoter-driven hygromycin phosphotransferase ( *HPT* ) gene and with the primer pair, GUS P2 plus GUS M10, to detect the auxin-inducible *mas* promoter-driven β-glucuronidase ( *GUS* ) gene. Additional confirmation was obtained by Southern blot analysis, performed according to Champagne and Ashton (2001), of *Bam* HI-digested LTII-2 genomic DNA, using as probe a 499 bp digoxygenin-labelled PCR amplicon derived from the *HPT* gene of plasmid pBDH001 cII. The Southern blot indicated the integration of tandem copies of the entire plasmid at two different locations in the genome. Final verification of the presence of pBDH001 cII in LTII-2 was demonstrated by the detection of uninduced and NAA-induced GUS expression in LTII-2 using the histochemical protocol of Jefferson et al. (1987).

## Reagents

**Table 1.** Primers used in this study.

**Table:** 

**Primer name**	**Sequence (5’ to 3’)**
HPT Upper	CGA TTC CGG AAG TGC TTG ACA TTG
HPT Lower	AAC CAC GGC CTC CAG AAG AAG ATG
GUS P2	CCT GTG GGC ATT CAG TCT GGA TCG
GUS M10	GGC AAT AAC ATA CGG CGT GAC ATC G

**Table 2.** Light filters used in this study.

**Table:** 

Filter name	Transmission range (nm)	Transmission maximum (nm)	Source
Hamburg Frost	280 - >850	Plateau at ~ 750-850	Rosco, 52 Harbor View Ave, Stamford, USA
Medium Red	565 - >850	Plateau at ~750-850	Rosco, 52 Harbor View Ave, Stamford, USA
Winter Blue	<400 - 510	445	Ruggieri Lighting and Staging Ltd., Regina, SK, Canada
